# Rosai-Dorfman disease in the breast: a case report and literature review

**DOI:** 10.3389/fonc.2026.1844736

**Published:** 2026-07-08

**Authors:** Manqing Cao, Liang Deng, Yuanyuan Sun, Yanhui Zhang, Liangsheng Liu, Hong Liu, Tong Wang

**Affiliations:** 1 The Second Surgical Department of Breast Cancer, Tianjin Medical University Cancer Institute & Hospital, National Clinical Research Center for Cancer, Tianjin, China; 2 Tianjin’s Clinical Research Center for Cancer, Key Laboratory of Breast Cancer Prevention and Therapy, Tianjin Medical University, Ministry of Education, Key Laboratory of Cancer Prevention and Therapy, Tianjin, China; 3 Department of Breast and Thyroid Surgery, The People's Hospital of the Qiandongnan Miao and Dong Autonomous Prefecture, Guizhou, China; 4 Department of Breast Pathology and Lab, Tianjin Medical University Cancer Institute & Hospital, Tianjin, China; 5 Department of Pathology, Tianjin Medical University Cancer Institute & Hospital, Tianjin, China; 6 Department of Breast Imaging, Tianjin Medical University Cancer Institute & Hospital, National Clinical Research Center for Cancer, Tianjin, China

**Keywords:** breast, extranodal involvement, lymph nodes, Rosai-Dorfman disease, surgery

## Abstract

Rosai-Dorfman Disease (RDD) is a rare form of sinus histiocytosis characterized by massive lymphadenopathy, commonly involving lymph nodes and occurring more frequently in children and young adults. Extranodal involvement of the breast is particularly uncommon. Here, we report a rare case of breast RDD, detailing the clinical findings, diagnostic process, and treatment, accompanied by a review of relevant literature. Given the limited cases of breast RDD reported, there is no established consensus on its diagnosis and treatment. This article aims to enhance understanding of breast RDD and inform clinical management practices.

## Introduction

RDD disease is a rare nonmalignant disease first discovered and proposed by French pathologist Pierre Paul Louis Lucien Destombes in 1965 (1), which is mainly caused by benign hyperplasia of sinus histiocytes, which mainly involves the lymph nodes and is also known as sinus histiocytosis with giant lymphadenopathy (2). While Rosai-Dorfman disease typically presents with cervical lymphadenopathy, extranodal involvement occurs in up to 43% of cases (3). Isolated extranodal disease is less common, reported in approximately 23% of patients (4). It often occurred in skin, soft tissues, sinuses, eyes, bone, central nervous system, urinary system, gastrointestinal system and breast (5), with breast involvement being sporadic, usually presenting as a painless palpable mass, either unilaterally or bilaterally (6, 7). It has been reported that the location of the lesion is mainly in the subcutaneous layer rather than in the parenchymal breast tissue, suggesting that the lesion originates in the dermis (8), which consistent with the preference of cutaneous RDD. Breast RDD disease occurring more often in women over 50 years of age, which may be related to regular screening for breast cancer in women over 50 years old (9). The etiology and pathogenesis are still unclear, but some studies suggest that RDD is a disease caused by autoimmune regulatory disorders and infections, including autoimmune hemolytic anemia, systemic lupus erythematosus, immune arthritis, and glomerulonephritis, etc., and may be associated with viral infections such as herpesvirus, EBV, cytomegalovirus, or HIV (10, 11). In recent years, it has also been found that gene mutations may be responsible for the development of the disease, including mutations in *ARAF, MAP2K1* (12), *NRAS* and *KRAS* (13, 14). Among them, the most frequent mutations involve KRAS and MAP2K1, often occurring in a mutually exclusive manner (13), and have been observed in up to one-third of RDD patients. Mutations in MAP2K1 have also been identified in isolated cases (14), and this accumulating evidence has been incorporated into the RDD consensus guidelines (15), which suggests that the *RAS/MAP2K1* pathway may be an essential component in the development of the disease and may be a potential therapeutic target (16), as well as a possible focus for future prospective studies on breast RDD disease (17).

## Case report

A 69-year-old woman visited our institution complaining of a self-detected palpable area of in the right breast and with gradually increasing in size over the past year. There is no nipple erosion, itching, overflow, no pain, or other self-conscious symptoms. She had a history of hypertension and cerebral infarction. She underwent bilateral knee arthroplasty with preoperative blood transfusion without transfusion reaction in 2014. Physical examination: There was a mass palpable at 5 cm from the nipple on the right breast, about 4 cm in diameter, hard, with unclear borders and poor mobility. There was no palpable mass in the left breast. No apparent enlarged lymph nodes were detected in the axillae and bilateral supraclavicular region. Normal development of both breasts, no erythema or cellulite-like changes in the skin of both breasts, no inversion of both nipples.

**Figure 1:**
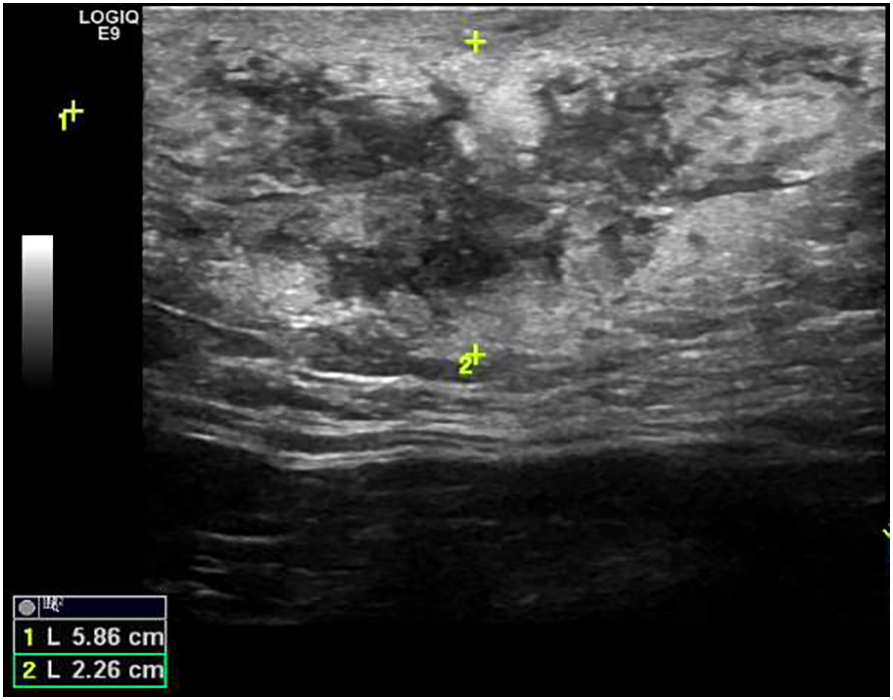
Ultrasound image of a mixed echogenic lesion in the upper outer quadrant of the right breast (5.9×5.1×2.3 cm), with indistinct margins, skin proximity, and abundant vascularity.

**Figure 2:**
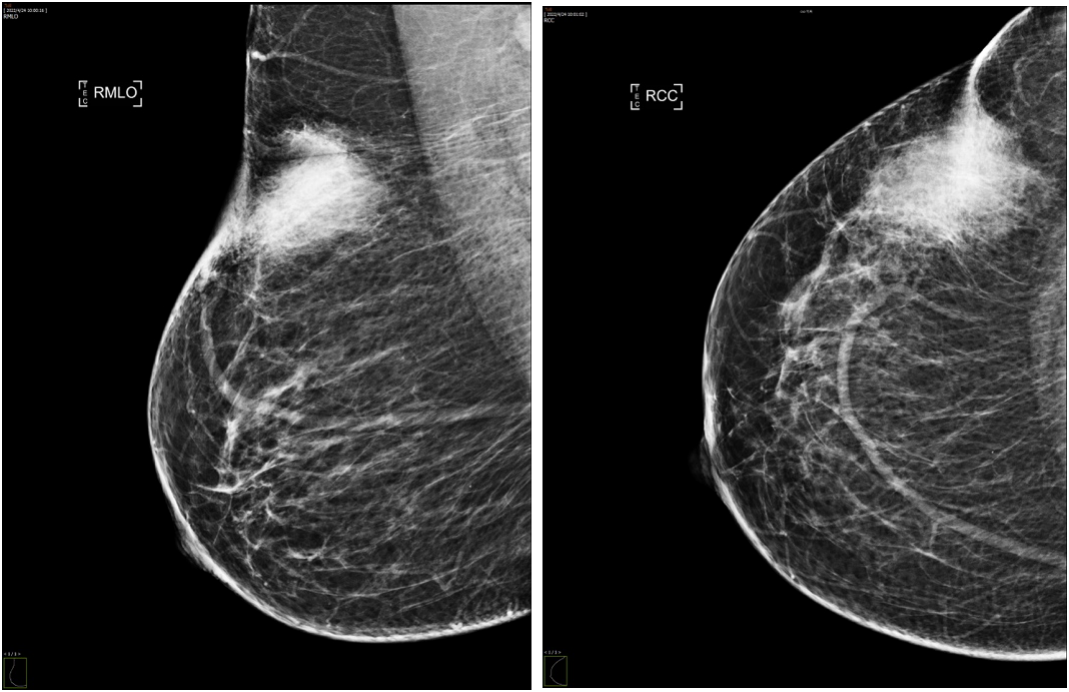
Mammography of the right breast showing a high-density mass in the upper outer quadrant with indistinct margins, corresponding to the palpable lesion.

Ultrasound revealed a mixed echogenic area in the right upper outer quadrant of the breast adjacent to the glandular margin, suggestive of a malignant lesion (**Figure 1**). Mammography revealed a dense lesion in the upper outer quadrant of the breast, suspicious for malignancy (**Figure 2**).

## Diagnostic process

An ultrasound-guided core needle biopsy was performed. The initial pathological report indicated that small cell lesions could not be excluded and recommended immunohistochemistry (IHC) for further diagnostic assistance. Subsequent IHC results of the core needle biopsy specimens were suggestive of an inflammatory lesion, and a comprehensive pathology consultation was advised to further rule out a lesion of mesenchymal origin. Then the right breast mass was subjected to wide local excision.

## Pathologic diagnosis

The morphology and immunophenotype of the lesion in the upper outer quadrant of the right breast, approximately 5 cm from the nipple, were consistent with extranodal Rosai-Dorfman disease. The lesion was in the dermis and subcutaneous tissue. Immunohistochemistry: CD20 (-), CD3 (-), S100 (+), CD68 (+), CD163 (+), CD1a (-), CD4 (-), Langerin (-), LCA (+), CD13 (partially+), MPO (-), Lysozyme (partially +), Ki67 (about 5%+), IgG (few scattered+), IgG4 (individual+), Kappa: Lambda was about 1-2:1.

## Outcome and follow-up

The patient was followed up for 48 months after complete surgical excision.

of the lesion, and no clinical recurrence (local recurrence or reoccurrence elsewhere) was observed.

### Preoperative breast ultrasound pictures

An area of approximately 5.9*5.1*2.3 cm of low to moderate mixed echogenic reflectivity was seen in the right breast, upper outer quadrant, approximately 5 cm from the nipple, with indistinct boundaries, close relationship with the skin, uneven internal echogenicity, and coarse abundant blood flow signal.

### Preoperative mammogram picture

**Figure 2** Mammography of the right breast, upper outer quadrant, approximately 5 cm from the nipple, in the mediolateral oblique and craniocaudal projections, demonstrates a high-density mass with indistinct margins. The mass corresponds to the patient-identified sites of palpable concern.

### Postoperative pathology images

**Figure 3:**
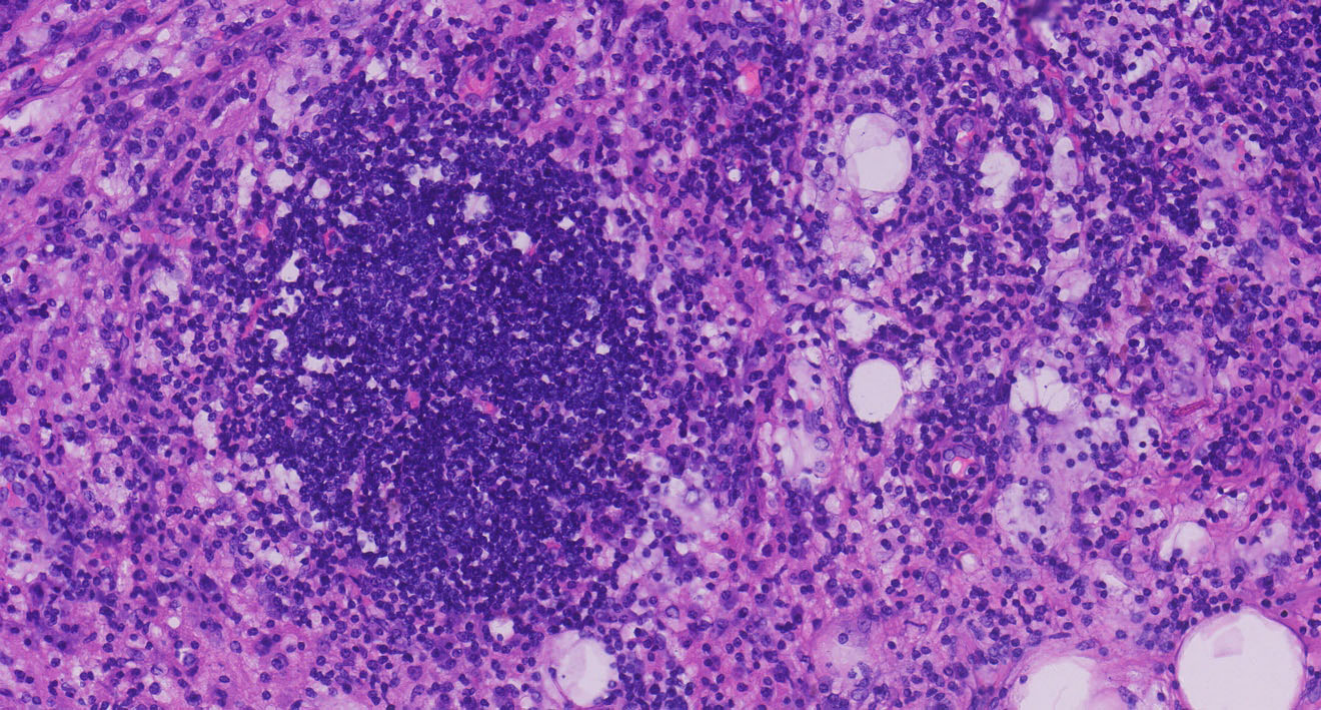
H&E staining of breast RosaiDorfman disease showing emperipolesis (scale bar = 50 µm).

Haematoxylin and eosin (H&E) staining of breast Rosai–Dorfman disease (scale bar = 50 µm) (**Figure 3**). The lesion is composed of sheets of large histiocytes with pale eosinophilic cytoplasm and vesicular nuclei. Admixed plasma cells and lymphocytes are present. Notably, some histiocytes exhibit emperipolesis (lymphocytes engulfed within the cytoplasm), a diagnostic hallmark of RDD.

**Figure 4:**
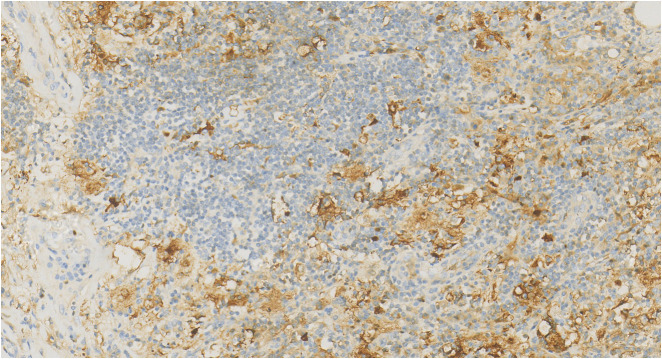
S-100 immunohistochemical staining of breast Rosai–Dorfman disease showing strong nuclear and cytoplasmic positivity in histiocytes, with admixed negative lymphocytes and plasma cells.

Rosai–Dorfman disease of the breast (S-100 immunohistochemical staining, original magnification ×20) (**Figure 4**). The histiocytes show strong cytoplasmic and nuclear positivity for S-100, which is a characteristic immunophenotype of RDD. Scattered positive cells are admixed with negative lymphocytes and plasma cells, highlighting the diagnostic histiocytic population.

**Figure 5:**
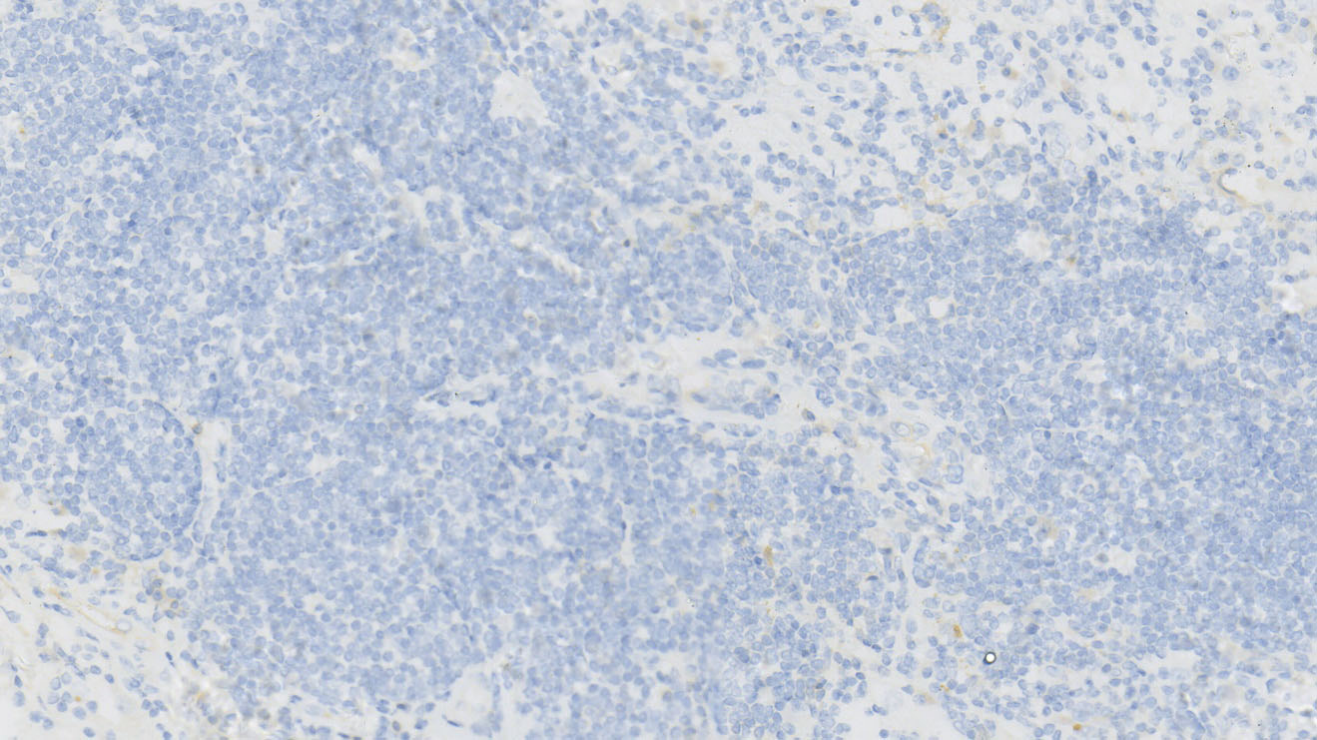
CD1a immunostaining of breast Rosai–Dorfman disease shows negative histiocytes with internal positive control lymphocytes (scale bar = 50 µm).

Breast Rosai-Dorfman disease (CD1a, scale bar = 50 µm) (**Figure 5**). The histiocytes show no CD1a expression (negative), with internal positive control lymphocytes present.

**Figure 6:**
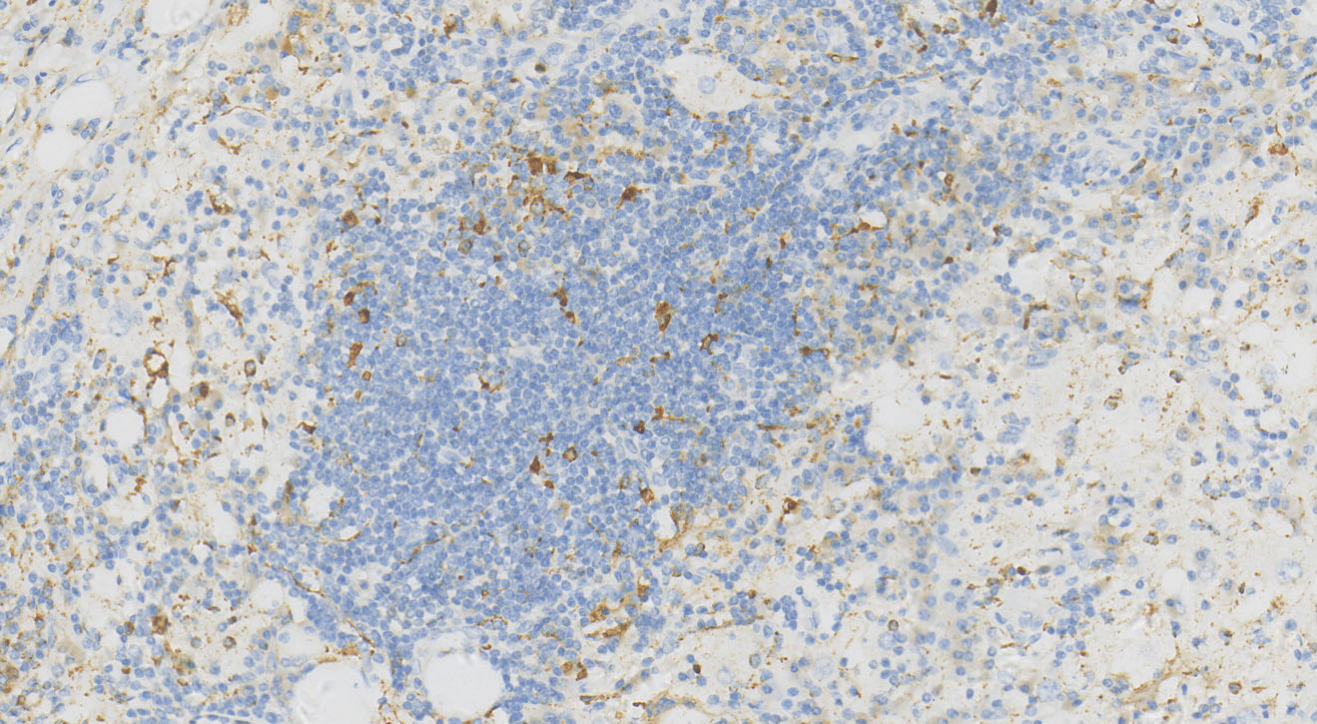
CD68 immunohistochemical staining of breast Rosai–Dorfman disease showing strong granular cytoplasmic positivity in histiocytes (scale bar = 50 µm).

Breast Rosai-Dorfman disease (CD68, scale bar = 50 µm) (**Figure 6**). The histiocytes exhibit strong granular cytoplasmic CD68 positivity, consistent with histiocytic differentiation.

## Summary of clinical and histopathologically findings

A total of 43 articles were searched in Pubmed using key word of “Rosai-Dorfman Disease” and “Breast” between 2013-2024. Studies that described RDD in other anatomical sites without breast involvement were excluded, and a total of 15 article involving 22 breast-related RDD cases were found as follows (**Table 1**):

**Table 1:** Clinical data of breast Rosai–Dorfman disease cases.

Author	Age	Gender	Breast laterality and mass size(cm)	Extramammary involvement	Radiological features (including BI-RADS)	Treatment	Immunochemistry(IHC)	FUp(month)	recurrence	Personal history
Liu, M et al(2018) (18)	65	Female	RB:1.8×1.5×1.5	No	*	excisional biopsy	S100(+++), IgG4+/172HPF, IgG4/IgG 82%, Serum IgG4 level 150mg/dl	40	No	high blood pressure
	78	Female	RB:3x2x2	No	PET/CT scan revealed a nodule in the right breast	surgical resection	S100(+++), IgG4+/118HPF, IgG4/IgG 53%, Serum IgG4 level 18mg/dl	39	No	chronic bronchitis
	53	Female	LB:1.5	No	*	surgical resection	S100(++), IgG4+/15HPF, IgG4/IgG10%	155	No	*
	45	Female	LB:*	No	*	surgical resection	S100(++), IgG4+/22HPF, IgG4/IgG 24%	86	No	type 2 diabetes
Sumner, C et al. (2023) (19)	59	Female	RB:1.4 LB:1.1	No	Mammography: a round mass in the RB and a focal asymmetry in the LB. Ultrasound images: an irregular solid mildly vascular mass with angular margins in the RB; Two irregular hypoechoic and hyperechoic solid masses in the LB	Close observation	S-100 (+), OCT2 (+), CD163(+)	*	*	Hypertension and Type II Diabetes
Delaney, EE et al. (2017) (4)	63	Female	RB:1.5	No	Mammogram: a focal asymmetry in the RB; Ultrasound: a solid mass with irregular borders and surrounding edema at 11 o’clock 9 cm from the nipple	No	strong and diffuse staining of S100, a subset of the stained with CD68	*	No	No
Nadal, M et al(2015) (20)	72	Female	RB: 9*6	No	Mammography and ultrasound imaging showed normal breast parenchyma	methotrexate	S100 (+), CD68 (+), CD163 (+), CD1a (-)	Follow-up 3 and 6 months later showed sustained partial response	*	Breast cancer family history
El-Attrache, B et al. (2017) (21)	55	Male	RB:3	No	Mammogram: sub-areolar mass in the RB with irregular margins. Ultrasound: an abnormal echo-texture inaninfiltrative pattern without a discretemass in the RB.	excision of the right breast mass	S100 (+)	25 (22)	*	Erectile dysfunction, dyslipidemia, glaucoma, vitamin D deficiency, hypertension, diabetes mellitus type 2, a history of deep vein thrombosis, and a prior inguinal hernia repair. Family cancer history included liver cancer in his father.
Zhou, Q et al. (2016) (23)	71	Female	LB: 0.5*0.3*0.5 (12 o’clock) and 0.4*0.4*0.3 (2 o’clock)	No	mammography: persistence of 2 new nodules within the LB. Ultrasonography: the presence of an oval parallel mass with slightly irregular margins at the 12:00 position, a hypoechoic nodule near the 2:00 position	A stereotactic core biopsy of the left breast and finally curative resection	S100 (+++), CD1a (-), CD68 (+), CD138 (+)	*	*	History of breast cancer s/p right total mastectomy, hypertension, hypothyroidism, and right knee osteoarthritis
Iancu, G et al. (2021) (24)	63	Female	RB:2.5 LB:1.5	systemic histiocytosis (bone located	Mammography: an opacity on the RB towards axillary tail with slightly irregular, and on the LB a similar image distributed at the border between inferior quadrants. Ultrasound imaging: BIRADS 4a. No suspicious axillary lymph nodes visualized	Bilateral ultrasound guided core needle biopsy and close observation	S100 (+), CD1a (+), CD68 (+), CD20(-), CD3(-)	12	The lesion disappeared at 6 months	renal cancer (post-surgery), and systemic histiocytosis (bone located)
Shin, GW et al. (2020) (9)	54	Female	RB:3.3	No	Ultrasonography: hypoechoic, ill-defined mass with an echogenic halo and increased internal vascularity in the RB. The imaging findings were suspicious for malignancy. There was no suspicious lymphadenopathy in both axillary areas Recurrence US: ill-defined hypoechoic mass with a hyperechoic halo and increased internal vascularity at the excision site	Surgical excision	S100 (+), CD68 (+)	6 months	6 months	Hypertension
Battle, B et al. (2021) (2)	49	Female	RB:1.4 × 0.8 × 1.1 (at 1:00); 2.7 × 1.5 × 2.5 (at 2:00); multifocal	No	Ultrasound: multiple similar appearing masses in the RB, including a hypoechoic, irregular mass at 1:00, as well as a hypoechoic, irregular mass at 2:00	ultrasound guided core needle biopsy	*	*	*	No
Shetty, S et al. (2020) (25)	54	Female	RB:6	No	Asymmetry; BI-RADS: 3	Partial mastectomy	S100 (+), CD68 (+), AE1/3(−)	168	No	*
	58	Female	RB: *	No	*	Excisional biopsy	S100 (+), CD68 (+), AE1/3(-)	*	*	*
	63	Female	RB: 2.2	axillary tail	BI- RADS: 4	Partial Mastectomy	S100 (+), CD68 (+), AE1/3(-), IgG4/IgG(-)	12	No	*
	52	Female	RB: 3	No	BI-RADS: 3	Breast reduction surgery	S100 (+), CD68 (+) CD3(-), CD20(-),	156	No	Macromastia
Choo, PZQ et al. (2021) (26)	52	Female	LB: *	No	Mammography: ill- defined density projected over the upper chest wall, sited a distance away from the breast fibroglandular tissue; Sonography: an oval mass with a thick hyperechoic rim and central hypoechoic area located in the subcutaneous fat, sited at the superior 12 o’clock location and medially adjacent to the axillary tail of the breast, BI-RADS: not specified	Core needle biopsy followed by excisional biopsy	*	48	No	*
	37	Female	LB: *	No	Mammography: subcutaneous left breast mass at the peripheral 7 o’clock position; sonography: a thick, ill- defined hyperechoic rim of fat tissue surrounding the central hypoechoic mass, BI−RADS: not specified	Core needle biopsy	*	*	*	*
Goldbach, AR et al. (2019) (8)	44	Female	RB:3	abdominal wall	Mammogram: an irregular, indistinct, high‐ density mass within the breast parenchyma Ultrasound: an irregular, indistinct, hypoechoic mass with posterior acoustic shadowing; BI‐RADS 4.	Ultrasound-guided biopsy;right breast lumpectomy; abdominal wall mass excision	S100 (+), CD68(+), CD163 (+)	*	No recurrence; no additional sites of disease	Hypertension, migraines
de Mello Tucunduva, TC et al. (2019) (27)	55	Female	LB:2.3*1.5	No	Mammogram: an incidental asymptomatic irregular mass in the LB, with ill‐defined borders;Ultrasound: a solid hypoechoic nodule with irregular borders	Ultrasound−guided core biopsy; surgical excision of breast, leg, and breast skin	*	2	a subcutaneous lump in her right leg and previous breast biopsy site	No
Tan, GSL et al. (2023) (28)	50’s	Female	RB: *	No	Mammography: a new partially imaged nodular opacity in the RB;Sonographic: a microlobulated hypoechoic nodule with ill-defined margins. PET/CT: FDG−avid. BI−RADS: not specified; imaging suspicious for malignancy	Ultrasound−guided core biopsy; surgical resection	S100 (+)	12	No	Left breast cancer surgery
Ciurea, A et al. (2016) (29)	59	Female	RB:*	No	Mammography: 2 well-defined, homogeneous masses in the RB;Sonography: 2 superficial oval, well-defined masses, with hyperechoic internal septae, with an ultrasound appearance similar to some of the fibroadenomas;At color Doppler evaluation, with low flow velocity, both masses showed intense blood supply; both of the breast lesions were homogeneously stiff, with an elasticity score of 4.	core needle biopsy	S100 (+)	18	Twelve years later, two lesions appeared on the back	No

* indicates that the information was not mentioned in the original case report.

RB, right breast; LB, left breast; FUp, follow-up.

### Inclusion criteria

1. Histopathologically confirmed Rosai-Dorfman disease with breast involvement;
2. Sufficient clinical, imaging, or follow-up data were available for extraction.

### Exclusion criteria

1. Studies without breast involvement;
2. Articles with only an abstract and no full text;
3. Reviews, editorials, or conference abstracts without original patient data;
4. Duplicate publications.

### Data extraction

Two independent reviewers; extracted items included age, gender, breast laterality and mass size, extramammary involvement, radiological features, Treatment, immunochemistry, follow-up, recurrence and personal history.

## Discussion

In patients with a confirmed diagnosis of RDD, cross-sectional imaging, including computed tomography (CT) of the neck, chest, abdomen, and pelvis, is advocated to evaluate the extent of organ or lymph node involvement (15). In pediatric patients, a chest radiograph combined with ultrasonography of the neck and abdomen is preferred to minimize radiation exposure. Although some researchers have employed fluorine-18 fluorodeoxyglucose positron emission tomography (FDG-PET)/CT for initial staging where feasible (15, 30), there is currently no consensus regarding the added value of PET/CT in RDD. Whole-body magnetic resonance imaging (MRI) is generally recommended for children in lieu of CT scanning to further reduce radiation exposure (31).

On breast imaging, RDD lesions are indistinguishable from malignant tumors (28, 32). Mammographic findings may reveal solitary or multiple circumscribed dense masses without calcifications (33). Ultrasound typically demonstrates a hypoechoic mass with ill-defined margins, abundant vascular signals, and atypical imaging features resembling those of breast cancer (9). The sonographic appearance of breast RDD may mimic that of primary breast malignancy, typically presenting as a hypoechoic mass with a hyperechoic halo (2, 9). Reports on breast MRI for the diagnosis of breast RDD are scarce; however, some investigators have suggested that an intramammary mass exhibiting benign imaging features, an inflow-type enhancement curve, and low apparent diffusion coefficient values may be indicative of breast RDD on MRI (34).

Core needle biopsy retrieves only a small sample from the lesion periphery, which contains few characteristic histiocytes, thereby rendering a definitive diagnosis challenging. Only a limited number of reported breast RDD cases have been successfully diagnosed via core needle biopsy (35). Although core needle biopsy may occasionally provide a definitive diagnosis (36, 37), the high imaging similarity between breast RDD and breast cancer (2, 33) often leads clinicians to perform surgical excision to obtain a larger tissue sample for diagnostic confirmation, particularly when CNB results are inconclusive or discordant with imaging findings (38). Consequently, the diagnosis of breast RDD remains predominantly dependent on histopathological examination.

Extranodal RDD lesions typically exhibit pronounced fibrosis, with benign proliferation of histiocytes characterized by abundant cytoplasm and vesicular nuclei (5, 24). Histopathologically, these lesions resemble chronic inflammatory processes with fibrosis, accompanied by numerous plasma cells and evidence of phagocytosis of lymphocytes, plasma cells, or erythrocytes (37). Some lesions exhibit fibrous tissue hyperplasia arranged in a vague storiform pattern (39). Under high-power microscopy, characteristic RDD cells can be identified as large histiocytes with abundant pale to eosinophilic cytoplasm and large, round, vesicular nuclei. These cells are admixed with numerous plasma cells and lymphocytes, forming a dense lymphoplasmacytic infiltrate. The histiocytes frequently contain intact lymphocytes within their cytoplasm, a phenomenon termed emperipolesis (6). Immunohistochemically, RDD cells consistently express S100, CD68, and CD163, while remaining negative for CD1a (6). In the present case, only scattered IgG-positive cells and occasional IgG4-positive cells were observed, with a κ:λ ratio of approximately 1–2:1, which does not meet the diagnostic criteria for IgG4-related disease (IgG4-RD). Nevertheless, the relationship between RDD and IgG4-RD warrants further comment. Several studies have reported RDD cases with increased IgG4-positive plasma cells, a phenomenon sometimes referred to as a “pseudo-IgG4 pattern” (7, 18, 40). Whether this represents a true overlapping syndrome, a reactive phenomenon, or a distinct subset of RDD remains unclear. In the breast, where both RDD and IgG4-RD are rare, awareness of this potential overlap is critical to avoid misdiagnosis. Clinicians should therefore exercise caution when interpreting limited IgG4 positivity, as it may not signify true IgG4-RD but rather a secondary inflammatory response.

## Conclusion

The management of RDD has traditionally been guided by the clinical experience of individual centers. However, the first consensus multidisciplinary recommendations for the diagnosis and management of RDD, published by Abla et al. in 2018 (15), have since provided a standardized framework for clinical decision-making. According to this consensus, most patients with localized, asymptomatic, or non-life-threatening RDD can be managed conservatively with observation alone, whereas local therapies such as surgical excision or radiotherapy are recommended for resectable, symptomatic lesions. For patients with refractory, multifocal, or life-threatening disease, systemic therapy including corticosteroids, chemotherapy, or targeted agents may be warranted.

Applying these principles to the cases of breast RDD summarized in **Table 1**, we observe that the majority of management strategies align well with the consensus recommendations (15). Cases 1 and 2, which presented as solitary, asymptomatic breast masses, were appropriately managed by observation and follow-up, consistent with the guideline’s stance on limited disease. Cases 3 and 4, where patients had progressively enlarged or symptomatic lesions, underwent surgical excision, which is the recommended local therapy for resectable disease. Case 5, involving widespread extranodal involvement including bilateral breast lesions, required systemic corticosteroid therapy, a treatment modality endorsed by the consensus for refractory or multifocal disease (15). These examples illustrate that while no breast-specific guidelines exist, the general RDD consensus framework provides a sound basis for decision-making in breast RDD, and our case series confirms its applicability in this rare extranodal presentation.

Regular monitoring is recommended for patients diagnosed by core needle biopsy. Given the indolent course of breast RDD and the frequent absence of systemic symptoms, a non-interventional approach with regular follow-up has been advocated (24). However, the optimal imaging modality for routine monitoring and detection of recurrence at other sites remains to be established; Although PET/CT has been proposed for this purpose (31, 41), its routine use is limited by high cost and radiation exposure concerns.

Surgical intervention is indicated when core needle biopsy is non-diagnostic due to insufficient sampling, when tumor burden reduction is required, or when the patient strongly desires surgery. Surgical excision is also appropriate for solitary lesions, given the limited evidence of spontaneous regression (33). However, radical excision should be avoided in breast RDD (24).

The overall prognosis of breast RDD is favorable, with a benign clinical course. However, patients with solid organ involvement (e.g., lungs, kidneys, liver) have a poorer prognosis, and sporadic fatal cases have been reported (4, 10), Prognosis also correlates with the number of involved lymph node groups (10). No fatal cases directly attributed to isolated breast involvement have been reported. Recurrence and disease progression have been observed in breast RDD (24, 27). but the exact recurrence rate remains unknown due to the small number of cases, short follow-up periods, and the lack of large-scale prospective studies (25).

## References

[1] Rosai J Dorfman RF . Sinus histiocytosis with massive lymphadenopathy. A newly recognized benign clinicopathological entity. Arch Pathol. (1969) 87:63–70. 5782438

[2] Battle B McIntire P Babagbemi K Mema E . Extranodal multifocal Rosai-Dorfman disease of the breast: A case report. Clin Imaging. (2021) 71:49–51. doi: 10.1016/j.clinimag.2020.07.012 33171367

[3] Mar WA Yu JH Knuttinen MG Horowitz JM David O Wilbur A . Rosai-Dorfman disease: Manifestations outside of the head and neck. AJR Am J Roentgenol. (2017) 208:721–32. doi: 10.2214/ajr.15.15504 28140608

[4] Delaney EE Larkin A MacMaster S Sakhdari A DeBenedectis CM . Rosai-Dorfman disease of the breast. Cureus. (2017) 9:e1153. doi: 10.7759/cureus.1153 28503389 PMC5426819

[5] Mantilla JG Goldberg-Stein S Wang Y . Extranodal Rosai-Dorfman disease: Clinicopathologic series of 10 patients with radiologic correlation and review of the literature. Am J Clin Pathol. (2016) 145:211–21. doi: 10.1093/ajcp/aqv029 26803323

[6] Wang Q Bradley K Zhang M Li S Li X . Rosai-Dorfman disease of the breast: a clinicoradiologic and pathologic study. Hum Pathol. (2023) 141:30–42. doi: 10.1016/j.humpath.2023.08.009 37673345

[7] Chimatira R Wessels R . Extranodal Rosai-Dorfman disease with increased IgG4-positive plasma cells involving the breast: A case report with review of the literature. Am J Dermatopathol. (2026) 48:391–6. doi: 10.1097/dad.0000000000003195 41417463

[8] Goldbach AR Hava S Caroline D Zhao X Bains A Pascarella S . Rosai-Dorfman disease of the breast: A potential marker of systemic disease. Breast J. (2019) 25:134–7. doi: 10.1111/tbj.13169 30488580

[9] Shin GW Park YM Heo YJ Baek JW Lee YJ Han JY . Sonographic features of Rosai-Dorfman disease in the breast: A case report. J Clin Ultrasound. (2020) 48:108–10. doi: 10.1002/jcu.22781 31638720

[10] Foucar E Rosai J Dorfman R . Sinus histiocytosis with massive lymphadenopathy (Rosai-Dorfman disease): review of the entity. Semin Diagn Pathol. (1990) 7:19–73. doi: 10.1001/archderm.1988.01670080023011 2180012

[11] Bruce-Brand C Schneider JW Schubert P . Rosai-Dorfman disease: an overview. J Clin Pathol. (2020) 73:697–705. doi: 10.1136/jclinpath-2020-206733 32591351

[12] Diamond EL Durham BH Haroche J Yao Z Ma J Parikh SA . Diverse and targetable kinase alterations drive histiocytic neoplasms. Cancer Discov. (2016) 6:154–65. doi: 10.1182/blood.v126.23.481.481 PMC4744547 26566875

[13] Garces S Medeiros LJ Patel KP Li S Pina-Oviedo S Li J . Mutually exclusive recurrent KRAS and MAP2K1 mutations in Rosai-Dorfman disease. Mod Pathol. (2017) 30:1367–77. doi: 10.1038/modpathol.2017.55 28664935 PMC5837474

[14] Matter MS Bihl M Juskevicius D Tzankov A . Is Rosai-Dorfman disease a reactve process? Detection of a MAP2K1 L115V mutation in a case of Rosai-Dorfman disease. Virchows Arch. (2017) 471:545–7. doi: 10.1007/s00428-017-2173-4 28597077

[15] Abla O Jacobsen E Picarsic J Krenova Z Jaffe R Emile JF . Consensus recommendations for the diagnosis and clinical management of Rosai-Dorfman-Destombes disease. Blood. (2018) 131:2877–90. doi: 10.1182/blood-2018-03-839753 29720485 PMC6024636

[16] Jacobsen E Shanmugam V Jagannathan J . Rosai-Dorfman disease with activating KRAS mutation - Response to Cobimetinib. N Engl J Med. (2017) 377:2398–9. doi: 10.1056/nejmc1713676 29236635

[17] Elbaz Younes I Sokol L Zhang L . Rosai-Dorfman disease between proliferation and neoplasia. Cancers (Basel). (2022) 14(21):5271. doi: 10.3390/cancers14215271 36358690 PMC9654168

[18] Liu M Li X Li Y Wang Z Cheng L Song X . Rosai-Dorfman disease with features of IgG4-related disease in the breast: Cases report and literature review. Asian Pac J Allergy Immunol. (2018) 36:51–7. doi: 10.1016/b978-1-4160-9976-5.10358-x 28577522

[19] Sumner C Salem K Abunimer L Ewaz A Zhang L Monsrud A . Bilateral breast Rosai-Dorfman disease screen detected by mammography. Clin Case Rep. (2023) 11:e6983. doi: 10.1002/ccr3.6983 36950663 PMC10025253

[20] Nadal M Kervarrec T Machet MC Petrella T Machet L . Cutaneous Rosai-Dorfman disease located on the breast: Rapid effectiveness of methotrexate after failure of topical corticosteroids, acitretin and thalidomide. Acta Derm Venereol. (2015) 95:758–9. doi: 10.2340/00015555-2057 25632986

[21] El-Attrache B Kapenhas E Morgani J Ahmed T . A rarity in breast pathology: A male case of Rosai-Dorfman disease and literature review. Int J Surg Case Rep. (2017) 37:1–3. doi: 10.1016/j.ijscr.2017.05.011 28600957 PMC5466548

[22] El-Attrache B Gluck B Heimann A Kapenhas E . A rarity in breast pathology: First recurrent male case of Rosai-Dorfman disease. Int J Surg Case Rep. (2018) 52:137–9. doi: 10.1016/j.ijscr.2018.10.003 PMC6199774 30359898

[23] Zhou Q Ansari U Keshav N Davis F Cundiff M . Extranodal manifestation of Rosai-Dorfman disease in the breast tissue. Radiol Case Rep. (2016) 11:125–8. doi: 10.1016/j.radcr.2016.04.012 27594932 PMC4996911

[24] Iancu G Gica N Mustata LM Panaitescu AM Vasile D Peltecu G . Rosai-Dorfman disease: Breast involvement-case report and literature review. Med (Kaunas). (2021) 57(11):1167. doi: 10.3390/medicina57111167 34833385 PMC8624438

[25] Shetty S Sharma N Booth CN Oshilaja O Downs-Kelly EP McKenney JK . Mammary extranodal Rosai-Dorfman disease with and without associated axillary lymphadenopathy: Insights for practitioners of breast pathology. Int J Surg Pathol. (2020) 28:541–8. doi: 10.1177/1066896920901770 31992097

[26] Choo PZQ Loh AHL Selvarajan S Tan PH Tan VKM Yong WS . Breast-related extranodal Rosai-Dorfman disease presenting as subcutaneous masses with thick hyperechoic rim, with review of the literature. Breast J. (2021) 27:883–6. doi: 10.1111/tbj.14286 34467595

[27] de Mello Tucunduva TC Gaziero A Tostes VS Chaves MC Stiepcich MMA Torres US . Extranodal Rosai-Dorfman disease manifesting with breast involvement: Imaging and histopathological findings. Breast J. (2019) 25:1266–7. doi: 10.1007/978-3-031-98844-8_27 31290189

[28] Tan GSL Chan CW . Rosai-Dorfman disease of the breast: the breast cancer mimic. BMJ Case Rep. (2023) 16(4):e255378. doi: 10.1136/bcr-2023-255378 37045551 PMC10105984

[29] Ciurea A Ciortea C Cosarca M Rogojan L . Breast involvement in pure cutaneous Rosai-Dorfman disease: Ultrasound and sonoelastography appearance with a review of the literature. Ultrasound Q. (2016) 32:183–6. doi: 10.1097/ruq.0000000000000170 26998930

[30] Mahajan S Nakajima R Yabe M Dogan A Ulaner GA Yahalom J . Rosai-Dorfman disease-utility of 18F-FDG PET/CT for initial evaluation and follow-up. Clin Nucl Med. (2020) 45:e260–6. doi: 10.1097/rlu.0000000000003014 32349088 PMC8177955

[31] Lu X Wang R Zhu Z . The value of (18)F-FDG PET/CT in the systemic evaluation of patients with Rosai-Dorfman disease: a retrospective study and literature review. Orphanet J Rare Dis. (2023) 18:116. doi: 10.1186/s13023-023-02711-8 37179326 PMC10182668

[32] Yu KK Briones NF Chan M Ahmed A Stevens E . Rosai-Dorfman disease simulating metastatic breast carcinoma. JAAD Case Rep. (2019) 5:372–4. doi: 10.1016/j.jdcr.2019.02.021 31008172 PMC6454097

[33] Zamora KW Zalasin S Desai A Guo H . Rosai-Dorfman disease of the breast: Radiologic-pathologic correlation. J Breast Imaging. (2025) 7:576–91. doi: 10.1093/jbi/wbaf041 41145154

[34] Li H Li D Xia J Huang H Jiao N Zheng Z . Radiological features of Rosai-Dorfman disease: case series and review of the literature. Clin Radiol. (2022) 77:e799–805. doi: 10.1016/j.crad.2022.07.008 36038400

[35] Bouhani M Kamoun S . Extranodal Rosai-Dorfman disease of the breast presenting as inflammatory breast cancer. Balkan Med J. (2025) 42:565–6. doi: 10.4274/balkanmedj.galenos.2025.2025-8-9 40855717 PMC12576484

[36] Parkin CKE Keevil C Howe M Maxwell AJ . Rosai-Dorfman disease of the breast. BJR Case Rep. (2015) 1:20150010. doi: 10.1259/bjrcr.20150010 30363205 PMC6159159

[37] Pham CB Abruzzo LV Cook E Whitman GJ Stephens TW . Rosai-Dorfman disease of the breast. AJR Am J Roentgenol. (2005) 185:971–2. doi: 10.2214/ajr.05.0224 16177417

[38] Ingole S Pavithra V Sundaram S Dennis Joseph L Hussain SA Dev B . The false alarm: Rosai–Dorfman disease of breast: case report. Egypt J Radiol Nucl Med. (2022) 53:1–6. doi: 10.1186/s43055-022-00731-1 38164791

[39] Levine EA Landry MM . Rosai Dorfman disease of soft tissue. Surgery. (1994) 115:650–2. doi: 10.5858/arpa.2021-0116-RA 8178266

[40] Zhang X Hyjek E Vardiman J . A subset of Rosai-Dorfman disease exhibits features of IgG4-related disease. Am J Clin Pathol. (2013) 139:622–32. doi: 10.1309/ajcparc3yq0klioa 23596114

[41] Zhang J Cui R Li J Cao X Luo Y . Characterizing Rosai-Dorfman disease with [(18)F]FDG PET/CT: a retrospective analysis of a single-center study. Eur Radiol. (2023) 33:6492–501. doi: 10.1007/s00330-023-09561-9 36971850

